# mSphere of Influence: Modifying an Old Method To Study RNA-Protein Interactions

**DOI:** 10.1128/mSphere.00600-19

**Published:** 2019-09-04

**Authors:** Calvin Tiengwe

**Affiliations:** a Department of Life Sciences, Imperial College London, London, United Kingdom

**Keywords:** AGPC, OOPS, RNA binding proteins

## Abstract

Calvin Tiengwe works on posttranscriptional gene regulation and iron homeostasis in the parasitic protozoan Trypanosoma brucei. In this *mSphere of Influence* article, he reflects on how the paper “Comprehensive identification of RNA-protein interactions in any organism using orthogonal organic phase separation (OOPS)” by Queiroz et al. (Nat Biotechnol 37:169–178, 2019, https://doi.org/10.1038/s41587-018-0001-2) influenced his research by providing a tool to capture RNA-protein complexes on a global scale using acid guanidinium thiocyanate-phenol-chloroform (AGPC), an old method hitherto applied for RNA, DNA, or protein purification.

## COMMENTARY

Starting an academic research career as an independent investigator comes with great enthusiasm and momentum to begin performing experiments. But this enthusiasm is often juxtaposed with several challenges, one of which is finding and assimilating relevant methodologies to investigate your biological questions of interest. Soon after setting up shop, I searched the literature to compare existing methodologies for purifying RNA-protein complexes *in vivo*. It became apparent that current methods rely on derivatives of cross-linking and pulldown experiments where live cells are first irradiated with UV at a defined wavelength (UV cross-linking) and lysed, and then associated RNA-protein complexes are captured by either oligo(dT)-coupled beads or (co)immunoprecipitations (IP). These methodologies have been widely applied to a variety of organisms but sparingly so for Trypanosoma brucei, a relatively well-studied and well-established experimentally tractable system. After many unsuccessful attempts to replicate these methods in T. brucei, a colleague of mine (James Budzak, Imperial College London) brought to my attention the article by Queiroz et al. published in *Nature Biotechnology* (1). In this paper, the authors coupled UV cross-linking and the classic one-step phenol-chloroform liquid-phase separation method to capture all RNAs and their associated RNA-binding proteome. By eliminating the oligo(dT) and IP steps for RNA capture, the new technique simplifies existing methods by reducing technical variations in downstream processing steps, improves efficiency and reproducibility, and highlights the need to always think critically about each step in a protocol; acid guanidinium thiocyanate-phenol-chloroform (AGPC)-based RNA extraction has been around for almost 40 years (2) but never before applied to purifying RNA-protein complexes.

Acid guanidinium thiocyanate-phenol-chloroform (AGPC) is a widely used reagent for isolation of RNA (or in some cases DNA and proteins) from cells. It relies on the ability of macromolecules (RNA, DNA, and protein) to partition between distinct liquid phases in a single-step reaction. Once added to cells, upon centrifugation, the mixture separates into a lower organic layer, an interphase, and an upper aqueous phase containing RNA. Despite its wide application as a routine laboratory technique for separating these macromolecules, the interface has always been excluded in RNA preparations. In this paper, Queiroz et al. reasoned that prior to lysis with AGPC, irradiating cells with UV at 254 nm will result in accumulation of RNA-protein complexes at the interface. Several rounds of AGPC separation significantly enrich exclusively for RNA-protein adducts that can be methanol precipitated and further applied to a variety of downstream applications such as next-generation sequencing or proteomics. In this work, a well-established classical method was thus repurposed and optimized for capturing and identifying the complete RNA-bound proteome of three human cell lines and the bacterium Escherichia coli. In all cases, orthogonal organic phase separation (OOPS) allowed the authors to identify entirely novel RNA-binding proteins (RBPs) not captured by other methods. The new method is efficient, easily reproducible, and less expensive, and its broad application in any organism is particularly attractive for my research in that it could be adopted to identify novel posttranscriptional regulatory factors of iron deficiency in T. brucei. This is potentially interesting for trypanosomes because control of gene expression is posttranscriptional, relying almost exclusively on the temporal dynamics, activities, and functions of RBPs.

As it is often the case when one first attempts to dissect complex processes in any organism, there are bound to be setbacks, especially when trying to develop new or reproduce existing methodologies. In the search for iron regulatory proteins in T. brucei, we had initially identified an RBP and hypothesized that it was a key posttranscriptional regulator of iron uptake genes. Functional characterization of this RBP showed that its role in iron regulation may be much more complex than we initially thought. Since our major goal is to identify regulatory RNAs or RBPs that respond to an iron stress signal, we had to go back to the drawing board to find an unbiased approach. OOPS is an important step forward in the way we think about studying RNA-protein interactions in T. brucei. Not only can we now capture the total RNA-interactome from this system (to the best of my knowledge, this has not yet been published), but overexpressing, knocking out, or silencing specific RNA-binding proteins could be potentially useful for dissecting subcomplexes within a specific pathway of interest. Using chemical agents to perturb the system, it is now possible to study in a dynamic fashion how regulatory RNAs, mRNAs, and their associated interactors respond to these agents or stimuli.

It is worth mentioning that since coming across this paper, alternative variants of this method have been published (3–5). It turns out that three of these groups (1, 3, 4) were independently working on the same idea and coordinated efforts to simultaneously submit to bioRxiv.
